# Review of "*Karyotypes of Parasitic Hymenoptera*" by Vladimir E. Gokhman

**DOI:** 10.1186/1756-3305-2-37

**Published:** 2009-08-13

**Authors:** Gavin R Broad

**Affiliations:** 1 Department of Entomology, the Natural History Museum, Cromwell Road, London SW7 5BD, UK

## Abstract

Review of Gokhman VE: Karyotypes of Parasitic Hymenoptera

## Review

This book is principally the summary of a large body of work, spanning nearly 25 years, by Vladimir Gokhman [1]. He has carved a niche in karyology of parasitoid wasps and this book has much to offer in the way of chromosome data. The findings are explicitly linked to phylogenetic hypotheses and we are presented with some interesting theories about the evolution of chromosome form and number within the Hymenoptera. Within the insect order Hymenoptera more has been published on the aculeate, i.e. stinging, ants, bees and wasps. Gokhman summarises this information in an extensive introduction. In fact, so much information is provided on the aculeates and the plant-feeding sawflies that I am not really sure why the title of the book is restricted to parasitic [sic] Hymenoptera. Nevertheless, the principal interest of the author lies with the parasitoid wasps, those species that develop at the expense of another insect host, sometimes with very intimate physiological interactions. Throughout the book (including the title), these wasps are referred to as 'parasitic Hymenoptera'. I dislike the term 'parasitic' when applied to these insects when 'parasitoid' is a much more informative term and draws attention to the fact that they are not classic parasites but are more like highly specialised predators. Anyway, there are many interesting facts to be gleaned, such as that haploid chromosome numbers for just one family, the ants (Formicidae) range from 1 to 60. You may be interested to know that the large clade of 'microhymenoptera', generally tiny species of parasitoid wasps, have a much reduced chromosome set compared to their larger relatives.

Given the taxonomically restricted scope of the book, presumably it is intended mainly to be read by hymenopterists, rather than cytogeneticists. Given this readership, it is a shame that there is very little in the way of explanation or, especially, illustration of karyological terms and features. But what are the main uses of the book? As the author states, although there are distinct, phylogenetically informative differences in modal numbers of chromosomes, it is difficult to define clades on discrete karyological characters, so the phylogenetic utility of such data seems limited. We still know far too little about the karyotypes of various small, enigmatic groups to be able to say anything meaningful about karyotype evolution in the early diversification of the parasitoid wasps. A large portion of the book is devoted to phylogenetic trends in karyotype, but the phylogenetic hypotheses are not derived from chromosome data. The phylogenies used are often not particularly robust. I see the more interesting results of karyological work being at the taxonomic level. The last chapter of the book details the species-level taxonomic insights that have resulted from the author's karyological work. Results such as these played an important role in drawing attention to the frequent presence of cryptic species in insects. Cryptic species are popping up everywhere now and it is clear that traditional morphotaxonomy has (understandably) underestimated true species diversity. This is where karyology has been most informative, in drawing attention to morphologically weakly separated sister species or cryptic species with no discernible morphological differences. Some good examples are given in this book. Clearly, karyology has played and still can play an important role in the discrimination of closely related species, but that particular baton has now been taken up by DNA sequencing. DNA barcoding is not mentioned by the author, who has a very optimistic prognosis for the future of karyological studies on parasitoid wasps. I do not share that enthusiasm. For example, the author states (p.76) that the inclusion of karyotype data will become a necessity for new species descriptions. There is no evidence for this. Instead, the sequencing of the Cytochrome Oxidase I (barcoding) gene is becoming routine. Not all labs or taxonomists have easy access to, or funding for sequencing facilities, but the costs of sequencing are dropping and it is becoming a much more accessible technique. The author's techniques are elegant and do offer different perspectives on, particularly, species-level taxonomic problems, but I doubt that they will be taken up routinely by many researchers.

And now for the gripes relating to the publisher. This book is very expensive. For £108 ($169 or €119.95) you get 81 pages of synthesis and discussion and 79 pages of appendices that summarise the existing literature (with a few new results included) and references. For this price, one would expect some good editing from the publishers but that is not the case. The text has been translated from the original Russian, but not very well. The writing is often ungrammatical and sometimes very difficult to understand. Surely some good text editing is a prerequisite when charging this sort of money for a book?

## Competing interests

The author declares that they have no competing interests.

## References

[B1] Gokhman VE (2009). Karyotypes of Parasitic Hymenoptera.

